# A Biomimetic Scaffold for Single-Stage Reconstruction of Load-Bearing Segmental Bone Defects

**DOI:** 10.34133/bmef.0321

**Published:** 2026-10-08

**Authors:** Jianzhong Bai, Dachuan Liu, Jiaying Li, Huan Wang, Guoping Liu, Youzhi Hong, Wenyu Duan, Jiaxu Shi, Hongtao Zhang, Bin Li, Jing Qu, Song Chen

**Affiliations:** ^1^ Medical 3D Printing Center, Orthopedic Institute, Department of Orthopedic Surgery, The First Affiliated Hospital, School of Basic Medical Sciences, Interdisciplinary Innovation Center for Nanomedicine, MOE Key Laboratory of Geriatric Diseases and Immunology, Suzhou Medical College, Soochow University, Suzhou, Jiangsu 215000, China.; ^2^ Department of Orthopedics, The Second Affiliated Hospital of Shandong First Medical University, Tai’an, China.; ^3^ Jiangsu Key Laboratory of Drug Discovery and Translational Research for Brain Diseases, The First Affiliated Hospital of Soochow University, Soochow University, Suzhou, Jiangsu 215123, China.; ^4^ Department of Cell Biology, School of Basic Medical Sciences, Medical School of Soochow University, Suzhou 215123, China.

## Abstract

**Objective:** This study aimed to develop a single-stage biomimetic scaffold that recapitulates hierarchical bone microenvironments for the repair of critical-sized segmental bone defects. **Impact Statement:** This scaffold integrates structural support and biological cues, eliminating secondary surgery and enabling load-bearing repair, thus advancing clinical translation. **Introduction:** The current Masquelet technique uses nondegradable spacers requiring 2 surgeries. A degradable scaffold with regenerative capacity could overcome this limitation. **Methods:** A porous brushite framework was combined with decellularized periosteal matrix and decellularized bone matrix to mimic periosteal, cortical, and medullary niches. In vitro bone marrow mesenchymal stem cell (BMSC) assays and an in vivo rat femoral critical-sized defect model assessed osteogenesis, angiogenesis, and biomechanics. **Results:** The scaffold enhanced BMSC adhesion, osteogenic, and angiogenic activities in vitro. In vivo, it promoted complete defect bridging, vascularized bone formation, direct integration, and biomechanical restoration. Histology revealed progressive scaffold replacement via accelerated endochondral ossification. **Conclusion:** This biomimetic scaffold effectively coordinates vascularization and bone regeneration, achieving functional single-stage repair of load-bearing segmental defects.

## Introduction

Critical-sized segmental bone defects (CSDs) remain one of the most significant challenges in orthopedic surgery [1,2]. Typically defined as defects exceeding the intrinsic regenerative capacity of bone, these lesions frequently arise following high-energy trauma, infection, congenital malformations, or tumor resection [3]. Unlike small fractures that heal spontaneously through coordinated bone remodeling, CSDs fail to regenerate without clinical intervention and are often associated with delayed union, nonunion, chronic disability, and even limb loss [4,5]. As global populations age and the incidence of complex musculoskeletal injuries continues to rise, the demand for effective strategies capable of restoring large skeletal defects has become increasingly urgent [6–8].

Among current clinical approaches, the Masquelet technique represents one of the most widely adopted reconstructive strategies for managing large segmental bone defects [9]. This procedure relies on a 2-stage protocol in which a polymethyl methacrylate spacer is first implanted into the defect to induce the formation of a vascularized membrane, followed several weeks later by a second operation involving spacer removal and the implantation of autologous bone grafts [10,11]. Although this approach has achieved encouraging clinical outcomes, its success is intrinsically dependent on staged surgical intervention. The requirement for a second operation inevitably increases patient morbidity, surgical complexity, infection risk, healthcare expenditure, and rehabilitation time [12]. Furthermore, the nondegradable spacer serves merely as a temporary placeholder and contributes little to functional regeneration [13]. Consequently, a major unmet clinical need is the development of a biodegradable implant capable of simultaneously providing immediate structural support, biological stimulation, and progressive replacement by native tissue, thereby enabling single-stage reconstruction without secondary surgical intervention [14,15].

The difficulty of achieving such a goal lies in the extraordinary complexity of native bone architecture [16]. Long bones are not homogeneous structures but highly organized biological systems composed of multiple functional compartments that cooperate to orchestrate skeletal regeneration [17]. The periosteum forms the outermost regenerative niche and contains abundant skeletal progenitor cells, vascular networks, and signaling molecules that initiate repair following injury [18,19]. Beneath the periosteum, the cortical bone provides the mechanical rigidity required for load transmission and structural stability [20]. In contrast, the cancellous and medullary compartments constitute a highly porous and biologically active microenvironment that supports vascular infiltration, cellular migration, hematopoiesis, and bone remodeling [21]. During natural fracture healing, these compartments interact dynamically through tightly coordinated osteogenic and angiogenic processes. Moreover, load-bearing bone regeneration is inherently governed by local mechanotransduction and tight osteogenic–angiogenic coupling, wherein functional scaffolds must relay physiological mechanical stimuli to recruit skeletal progenitors and foster functional vascular ingrowth [22]. Therefore, successful regeneration of critical-sized defects requires not only new bone formation but also reconstruction of the hierarchical regenerative microenvironment that characterizes native skeletal tissue [23–25].

Despite substantial advances in biomaterials and tissue engineering, most currently available bone substitutes fail to recapitulate this level of structural and biological complexity. Metallic implants and additively manufactured metal scaffolds provide excellent mechanical strength but suffer from modulus mismatch, stress shielding, limited bioactivity, and concerns regarding long-term integration [26]. Hydrogels exhibit favorable biodegradability and tunable architectures but often lack sufficient mechanical competence for load-bearing applications [27–29]. Calcium phosphate ceramics, including hydroxyapatite and β-tricalcium phosphate, possess excellent osteoconductivity but frequently exhibit excessive brittleness and limited degradation kinetics, restricting their application in demanding load-bearing scenarios [30,31]. More importantly, although many of these materials have demonstrated promising osteogenic potential, most remain fundamentally homogeneous constructs that inadequately reproduce the spatial organization of native bone tissues. As a result, they often fail to establish the coordinated osteogenic and angiogenic microenvironments required for complete regeneration of large skeletal defects.

An emerging strategy for overcoming these limitations is the integration of synthetic structural biomaterials with biologically derived extracellular matrix (ECM) components [32–34]. Synthetic materials provide the mechanical framework necessary for defect stabilization, whereas decellularized matrices preserve the biochemical complexity of native tissues and deliver endogenous regenerative signals. Among available calcium phosphate materials, brushite (dicalcium phosphate dihydrate [DCPD]) represents a particularly attractive candidate because of its unique combination of high compressive strength, osteoconductivity, and controllable biodegradation [35–37]. Unlike permanent metallic implants or slowly resorbing ceramics, brushite can gradually degrade and be replaced by newly formed bone, making it highly suitable for regenerative reconstruction. Our previous studies demonstrated that optimized brushite cement can achieve compressive strengths exceeding 60 MPa while maintaining favorable biological performance [35]. Nevertheless, brushite alone lacks the tissue-specific ECM cues necessary to direct vascularization, stem cell recruitment, and coordinated tissue remodeling.

Decellularized ECMs provide an effective means of restoring these missing biological functions [38]. Decellularized bone matrix (DBM) retains the structural and biochemical characteristics of native bone tissue, including collagen-rich architecture and endogenous osteogenic growth factors, thereby providing an osteoinductive environment conducive to skeletal regeneration. Similarly, decellularized periosteal matrix (DPM) preserves the key components of the periosteal niche that regulate progenitor cell activation, angiogenesis, and early bone healing [26]. Historically, diverse natural biomaterials have been applied in regenerative orthopedics due to their intrinsic biocompatibility and physiological recognition motifs [39]. Specifically, DPM provides a native fibrous collagenous architecture and localized signaling microenvironment that act as an osteoinductive and proangiogenic sleeve to guide peripheral vascularization and cortical bridging. Previous studies have demonstrated the regenerative potential of both DBM and DPM in various orthopedic applications [40,41]. However, these biological matrices lack sufficient mechanical integrity to bridge critical-sized defects independently and therefore require integration with load-bearing structural frameworks.

Inspired by the hierarchical organization of native long bone, we developed a hierarchical biomimetic scaffold that recapitulates the periosteal, cortical, and medullary compartments within a single integrated construct (Fig. 1). The scaffold combines an outer DPM, a load-bearing porous brushite framework, and a DBM-filled core to provide coordinated biological and mechanical functions. This design aims to support vascularized bone regeneration while undergoing progressive degradation and replacement by host tissue, thereby eliminating the need for implant removal. We evaluated the scaffold through comprehensive in vitro studies and a rat femoral critical-sized segmental defect model, demonstrating its potential as a biodegradable, off-the-shelf platform for the single-stage reconstruction of load-bearing segmental bone defects.

**Fig. 1.**
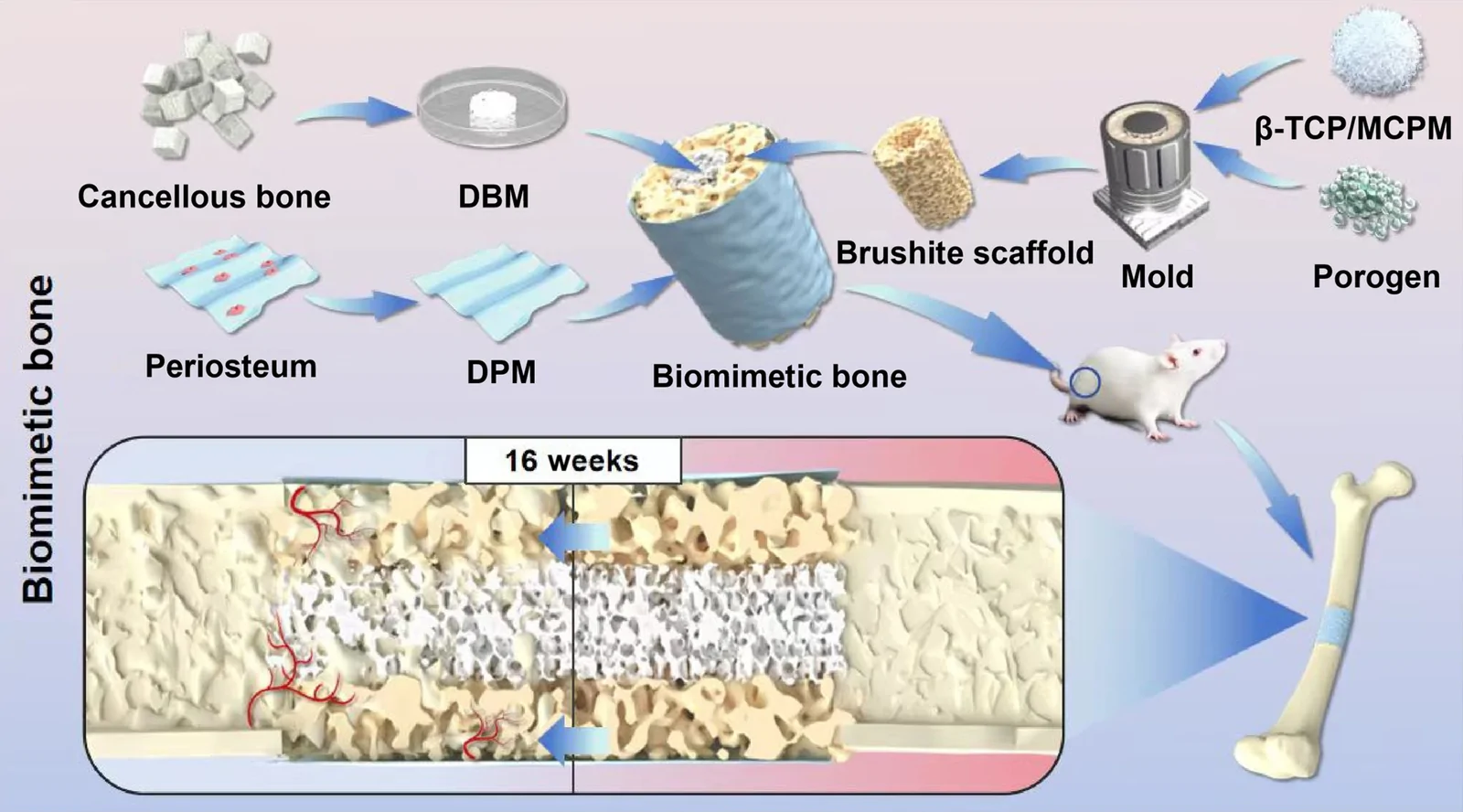
Design and application of biomimetic hierarchical scaffolds for segmental bone defect regeneration in a rat femoral defect model.

## Results

### Characterization of the integrated biomimetic scaffold

A hierarchical biomimetic scaffold that recapitulates the structural organization of native long bone was fabricated and characterized. Scanning electron microscopy (SEM) imaging revealed that the synthesized brushite exhibited a characteristic platelike crystalline morphology (Fig. 2A). The gelatin microspheres used as sacrificial porogens were spherical, with a diameter around 300 μm (Fig. 2B). Since increasing porosity is generally accompanied by a reduction in mechanical strength, different microsphere contents were evaluated to identify a suitable balance between structural integrity and pore formation. Micro-computed tomography (micro-CT) reconstruction demonstrated the formation of a homogeneous and highly interconnected pore structure within the scaffold (Fig. 2C). Quantitative analysis showed that scaffold porosity increased from 0.1% to 32.9% as the microsphere content increased from 0 to 15 wt% (Fig. 2D), whereas compressive strength decreased correspondingly (Fig. 2E). Considering the in vivo mechanical requirements of rat femoral defect repair, a microsphere content of 10 wt% was selected as the optimal formulation.

**Fig. 2.**
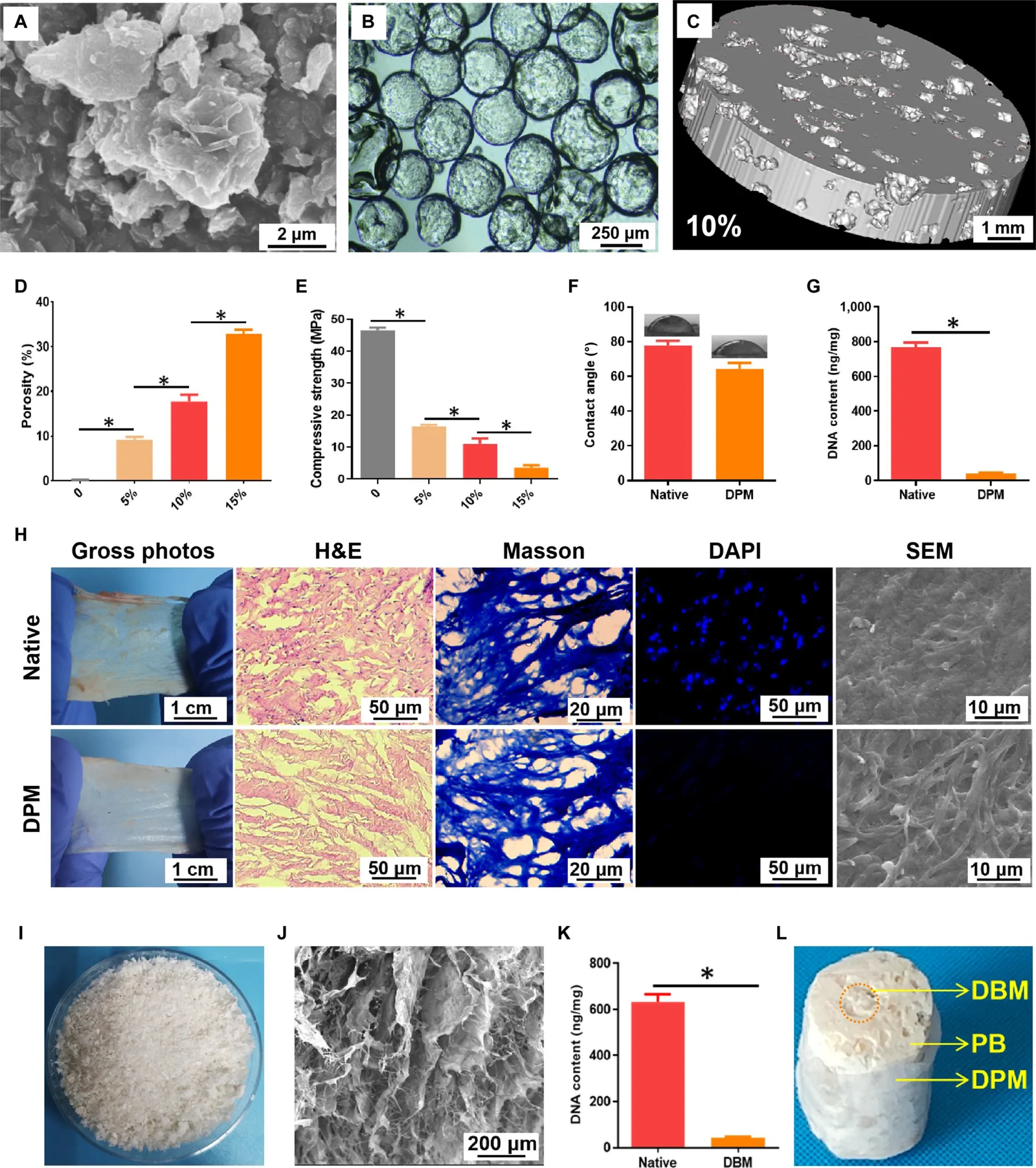
Design and characterization of the hierarchical biomimetic bone scaffold. (A) Scanning electron microscopy (SEM) image of the platelike brushite crystals. (B) Morphological characterization of gelatin microspheres. (C) Three-dimensional micro-computed tomography (micro-CT) reconstruction of the porous brushite scaffold. (D and E) Quantitative analysis of porosity and compressive strength with varying microsphere content. (F and G) Surface hydrophilicity (contact angle) and DNA quantification of DPM. (H) Characterization of DPM: physical flexibility; hematoxylin and eosin (H&E), 4′,6-diamidino-2-phenylindole (DAPI), and Masson staining; and SEM imaging. (I) Gross morphology of DBM. (J and K) Structural characterization and decellularization efficiency of DBM. (L) Schematic and macroscopic view of the integrated scaffold. DPM, decellularized periosteal matrix; PB, porous brushite; DBM, decellularized bone matrix. *P < 0.05.

The biological components of the scaffold were subsequently characterized. Following decellularization, DPM retained the flexibility and fibrous architecture of native periosteum (Fig. 2H). Histological staining confirmed the effective removal of cellular components, as evidenced by the absence of visible nuclei in both hematoxylin and eosin (H&E) and 4′,6-diamidino-2-phenylindole (DAPI) staining, while Masson’s trichrome staining demonstrated the preservation of the collagen-rich ECM. Consistent with these observations, SEM imaging revealed an intact fibrous network after processing (Fig. 2H). Decellularization also enhanced surface wettability, reducing the water contact angle from 77.8° ± 4.6° to 64.4° ± 5.7° (Fig. 2F). DNA quantification further confirmed efficient decellularization, with residual DNA levels reduced to 41.0 ± 4.2 ng/mg, below the commonly accepted threshold for decellularized biomaterials (Fig. 2G). Quantitative analysis revealed that collagen content showed no significant difference before and after decellularization in both DPM and DBM, confirming that the main structural protein matrix was well preserved. However, a significant loss of sulfated glycosaminoglycans was observed after processing (Fig. S1). Similarly, DBM exhibited a porous architecture closely resembling native cancellous bone, providing a favorable microenvironment for cell infiltration and nutrient exchange (Fig. 2I and J). DNA analysis verified effective cellular removal from the DBM (Fig. 2K). By integrating a DBM-filled core within the porous brushite framework and enclosing the construct with a DPM outer layer, a multizonal biomimetic scaffold was established. This hierarchical construct reproduces the periosteum–cortical bone–cancellous bone organization of native long bone and provides complementary structural and biological functions for subsequent regeneration studies (Fig. 2L).

### In vitro biocompatibility assessment of the biomimetic scaffold

The biocompatibility of the scaffold was evaluated using rat bone marrow mesenchymal stem cells (BMSCs) across 4 groups: blank control (Ctrl), porous brushite framework alone (PB), PB wrapped with DPM (PB/DPM), and the fully integrated construct (biomimetic bone, PB/DPM/DBM). SEM examination revealed extensive cell attachment throughout the porous brushite framework after 3 d of culture. Cells displayed a well-spread morphology with abundant pseudopodia extending along the pore walls and material surfaces, indicating favorable cell–material interactions (Fig. 3A to C). Consistent with these observations, cytoskeletal staining showed comparable cell spreading and F-actin organization in all scaffold extract groups relative to the untreated control (Fig. 3D). Live/Dead staining further demonstrated high cell viability across all groups, with survival rates exceeding 90% after 3 d of culture (Fig. 3E). Quantitative analysis confirmed the absence of detectable cytotoxicity associated with any scaffold component (Fig. 3F). Cell proliferation was subsequently assessed using the Cell Counting Kit-8 (CCK-8) assay. All scaffold extract groups exhibited a continuous increase in absorbance over the 5-d culture period, following proliferation kinetics similar to those of the control group (Fig. 3G). Together, these findings demonstrate excellent biocompatibility of the porous brushite framework, DPM, and DBM, supporting their application as bioactive components for bone regeneration.

**Fig. 3.**
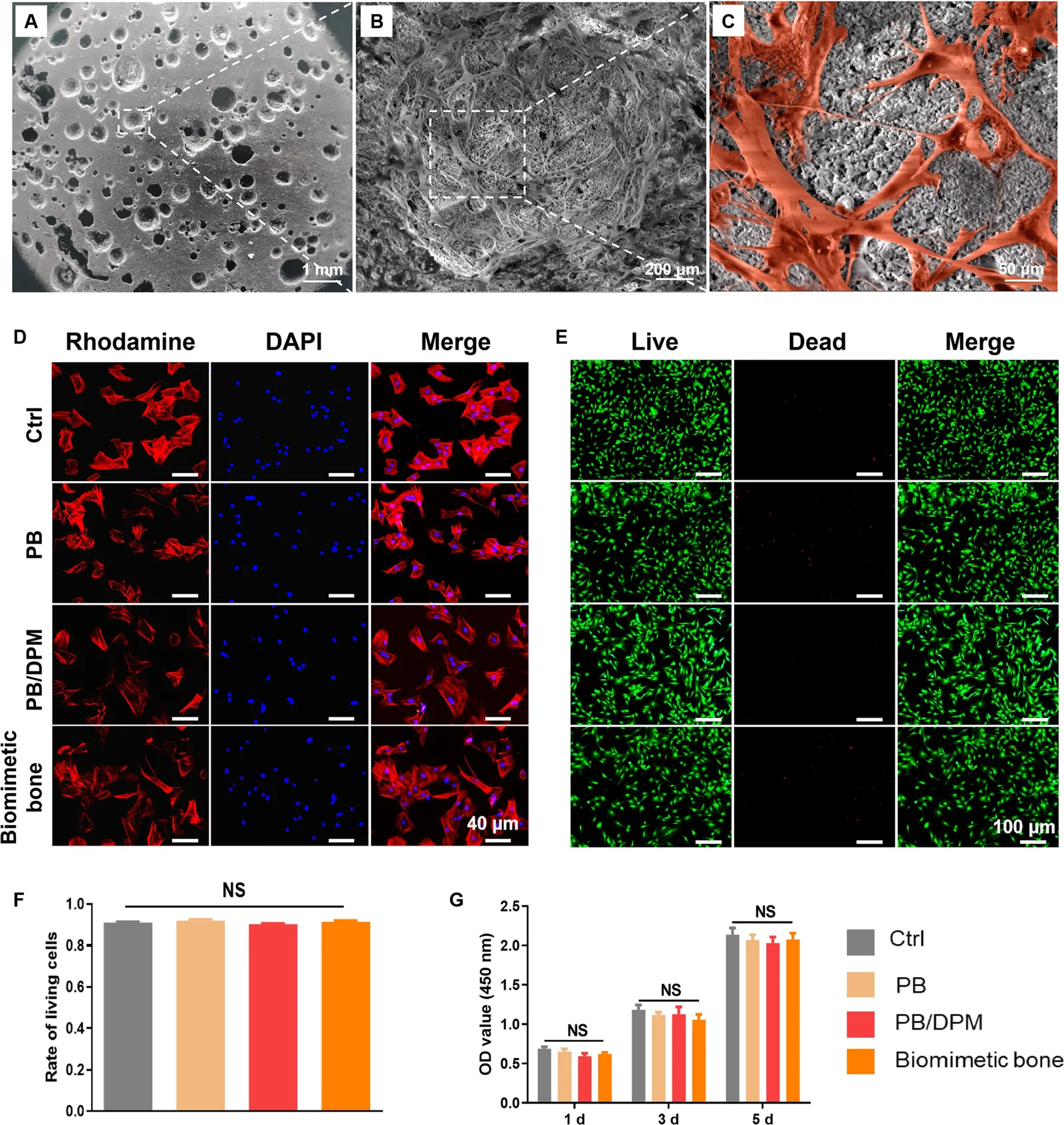
In vitro biocompatibility evaluation of the scaffold components. (A to C) Scanning electron microscopy (SEM) images showing bone marrow mesenchymal stem cell (BMSC) adhesion and pseudopodium extension on the scaffold. (D) Cytoskeletal staining of BMSCs cultured in different extract groups. (E and F) Live/Dead staining and corresponding quantitative cell viability at day 3. (G) Cell Counting Kit-8 (CCK-8) proliferation kinetics of BMSCs over 5 d. DPM, decellularized periosteal matrix; PB, porous brushite; DBM, decellularized bone matrix. NS, no significant difference.

### In vitro osteogenic activity of the hierarchical scaffold

The osteogenic potential of the different scaffold formulations was first evaluated by alkaline phosphatase (ALP) and Alizarin Red S (ARS) staining. After 7 d of culture, ALP staining intensity progressively increased from the PB group to the PB/DPM group and reached its highest level in the hierarchical biomimetic bone construct (Fig. 4A). A similar trend was observed after 14 d of culture, where ARS staining revealed the most extensive mineralized matrix deposition in the integrated biomimetic scaffold group (Fig. 4B). Quantitative analysis confirmed significantly greater ALP activity and calcium deposition in the biomimetic bone group compared with those in all other groups (Fig. 4C). To further investigate the underlying biological responses, osteogenic and angiogenic gene expression profiles were examined by reverse transcription quantitative polymerase chain reaction (RT-qPCR). All material groups significantly upregulated osteogenesis-related genes compared with the control group. Notably, the expression levels of alkaline phosphatase (*Alpl*), runt-related transcription factor 2 (*Runx2*), collagen type I alpha 1 chain (*Col1a1*), and osteopontin (*Opn*) were highest in the biomimetic bone group (Fig. 4D). In parallel, the angiogenic marker vascular endothelial growth factor A (*Vegfa*) was markedly elevated following the hierarchical integration of DPM and DBM, suggesting enhanced capacity to support vascularized tissue regeneration (Fig. S2).

**Fig. 4.**
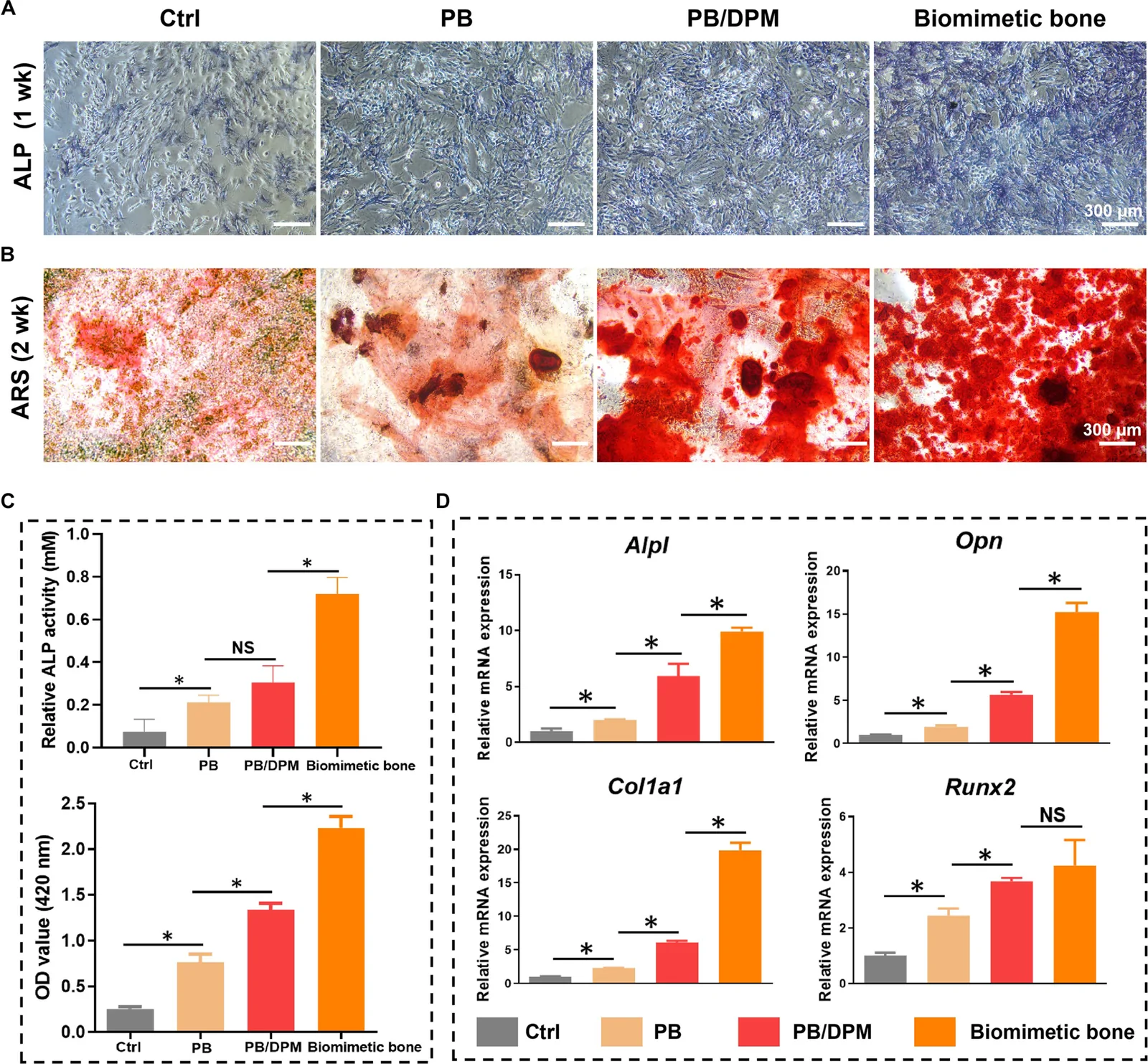
Osteogenic differentiation potential in vitro. (A) Alkaline phosphatase (ALP) staining of bone marrow mesenchymal stem cells (BMSCs) at 1 week. (B) Alizarin Red S (ARS) staining at 2 weeks. (C) Quantitative analysis of ALP and ARS staining. (D) Reverse transcription quantitative polymerase chain reaction (RT-qPCR) analysis of osteogenic genes (alkaline phosphatase [*Alpl*], osteopontin [*Opn*], collagen type I alpha 1 chain [*Col1a1*], and runt-related transcription factor 2 [*Runx2*]). *P < 0.05; DPM, decellularized periosteal matrix; PB, porous brushite; DBM, decellularized bone matrix. NS, no significant difference.

### Hierarchical biomimetic scaffolds enhance segmental bone regeneration in vivo

The regenerative performance of the different scaffolds was assessed using radiographic imaging and micro-CT. Schematic representations of the scaffold architectures and experimental groups are shown in Fig. 5A. At 16 weeks postimplantation, x-ray imaging revealed minimal spontaneous repair in the empty control group, characterized by persistent radiolucent defects (Fig. 5B). The solid brushite scaffold partially maintained structural continuity. However, bone regeneration was mainly confined to the defect margins, and a clear interface persisted between the scaffold and host bone. In contrast, the PB scaffold enabled pronounced bone ingrowth throughout the interconnected pore structure and promoted improved osseointegration. Notably, the incorporation of decellularized matrices further enhanced the regenerative response. Both the PB/DPM and biomimetic bone groups achieved complete bridging of the segmental defect, accompanied by extensive peri-implant bone formation and restoration of cortical continuity. Gross examination of the harvested femora confirmed the radiographic findings (Fig. 5C). Three-dimensional micro-CT reconstruction further visualized that the newly formed bone tissue on the material surface progressively increased from the solid brushite (SB) group to the biomimetic bone group (Fig. 5D). Quantitative analysis showed a stepwise increase in newly formed bone volume in the order of SB < PB < PB/DPM < biomimetic bone. Consistently, bone volume fraction (BV/TV) and trabecular number (Tb.N) were significantly increased by scaffold porosity and were further enhanced by the addition of DPM and DBM (Fig. 5E and F). In contrast, trabecular thickness (Tb.Th) showed no significant differences among groups (Fig. 5G). These results demonstrate that hierarchical scaffolds promote defect bridging and robust segmental bone regeneration.

**Fig. 5.**
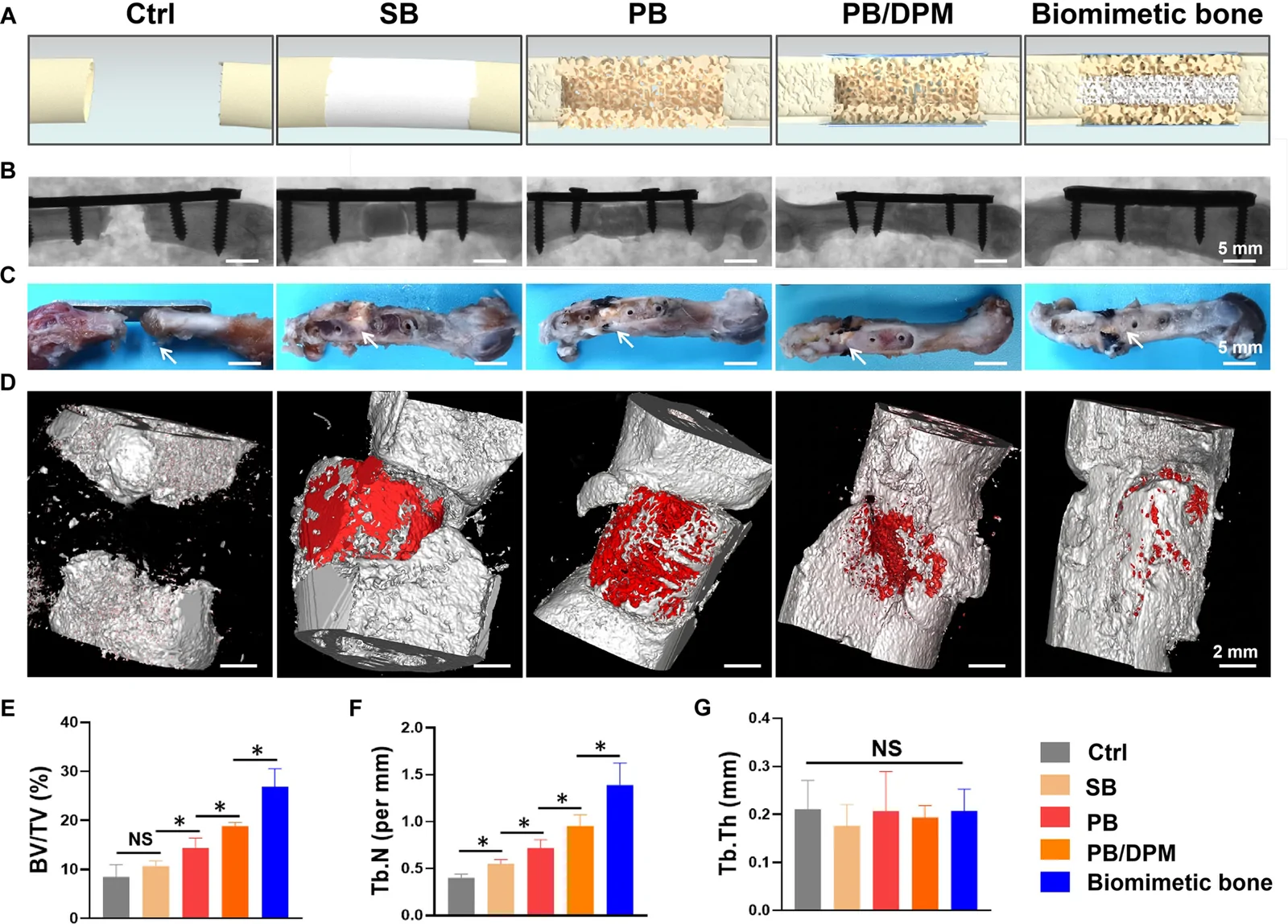
Radiographic and gross morphological assessment of bone repair at 16 weeks. (A) Schematic of the experimental groups in the rat femoral defect model. (B) Representative x-ray images showing defect bridging and osseointegration. (C) Gross morphology of harvested femora across all groups. White arrows delineate the original defect. (D) Three-dimensional (3D) micro-computed tomography (micro-CT) reconstructions of new bone tissue on the material surface (red, composite scaffold; white, new bone). (E to G) Quantitative analysis of micro-CT parameters, including bone volume fraction (BV/TV), trabecular number (Tb.N), and trabecular thickness (Tb.Th). SB, solid brushite; PB, porous brushite; DPM, decellularized periosteal matrix; DBM, decellularized bone matrix. *P < 0.05; NS, no significant difference.

### Hierarchical scaffolds restore load-bearing capacity in segmental bone defects

The successful repair of segmental bone defects requires not only bone formation but also the restoration of functional mechanical integrity. To evaluate osseointegration and load-bearing performance, biomechanical analyses were performed following defect healing. Although the PB/DPM scaffold was capable of supporting a transverse load of 0.5 kg, a noticeable deformation was observed at the implant–bone interface, indicating incomplete mechanical integration (Fig. 6A and B). In contrast, the biomimetic bone scaffold maintained structural stability under both transverse and longitudinal loading conditions (Fig. 6C to E). Quantitative mechanical testing further demonstrated superior fixation strength in the hierarchical scaffold group, with pullout forces exceeding 130 N and outperforming all other treatment groups (Fig. 6F and G). Microstructural examination revealed progressive degradation of the brushite framework accompanied by continuous ingrowth of newly formed bone throughout the scaffold architecture (Fig. 6H). SEM analysis revealed intimate contact between regenerated bone and scaffold surfaces, with no detectable interfacial gaps (Fig. 6I). Consistently, backscattered electron and SEM imaging revealed extensive mineralized tissue formation within the scaffold pores and direct structural continuity between host bone and regenerated tissue (Fig. 6J to L). These observations suggest a progressive scaffold-to-bone transformation process in which newly formed bone gradually replaces the degrading biomaterial, ultimately restoring mechanical competence across the defect site.

**Fig. 6.**
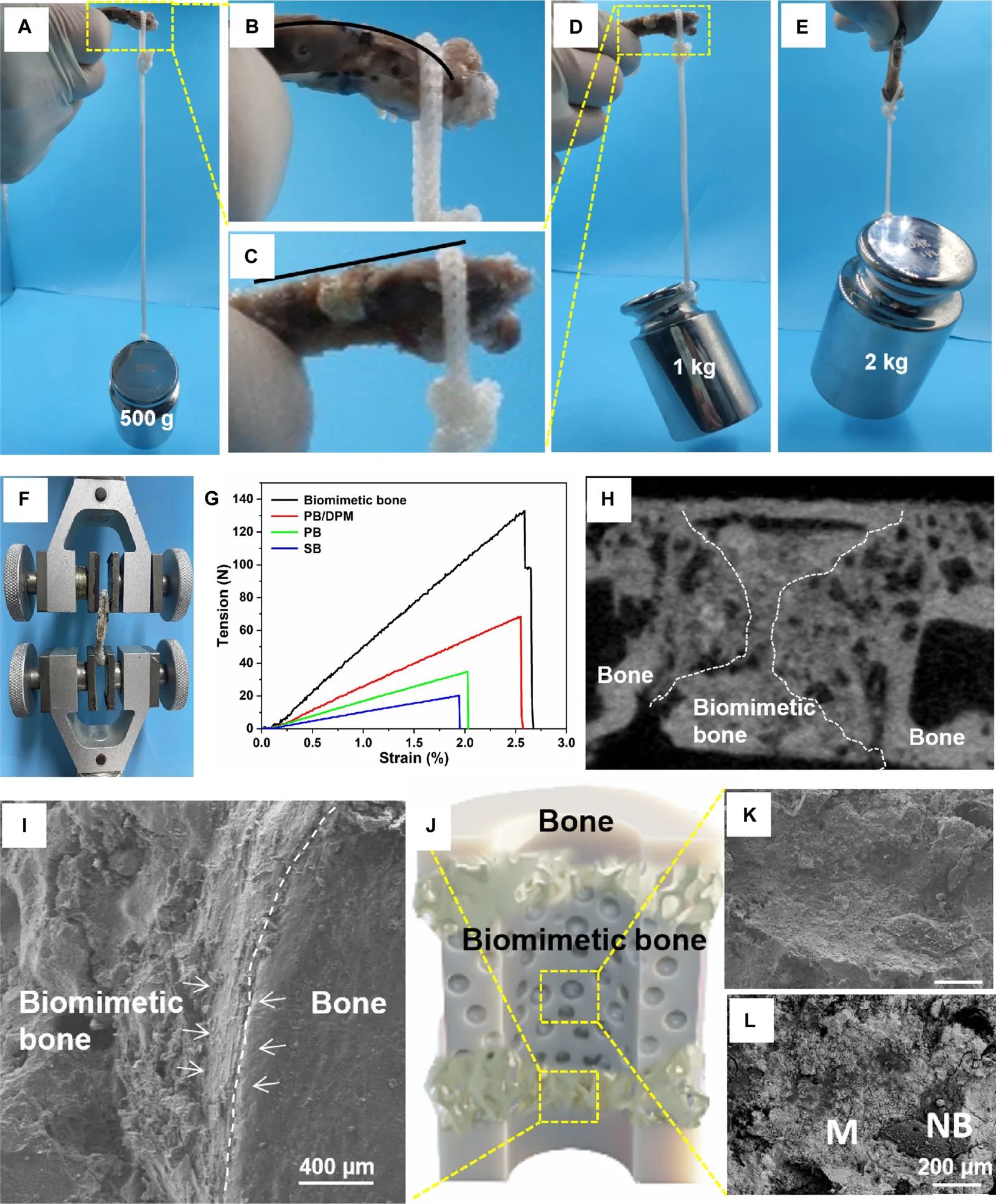
Biomechanical performance and microstructural characterization of scaffold–bone integration. (A and B) Deformation of the PB/DPM scaffold under a 500-g transverse load, showing interfacial instability. PB/DPM, porous brushite wrapped with decellularized periosteal matrix. (C to E) Mechanical stability of the hierarchical scaffold under (D) 1-kg transverse and (E) 2-kg longitudinal loading conditions. (F) Tensile strength testing setup and (G) representative stress–strain curve showing maximal pullout strength. (H) Micro-computed tomography (micro-CT) reconstruction illustrating progressive scaffold resorption and bone ingrowth at the interface (dashed lines indicate the approximate transitional boundary between host bone and the 5-mm defect area based on radiodensity and trabecular pore distribution). (I) Scanning electron microscopy (SEM) image confirming intimate, gap-free integration at the host bone–scaffold interface (delineated by matrix architecture and compositional contrast). (J) Schematic illustration of hierarchical scaffold–bone integration. (K and L) SEM and backscattered electron (BSE) images showing extensive intrascaffold osseous ingrowth (M, material; NB, new bone).

### Histological evidence of cartilage-mediated bone regeneration

H&E staining at 16 weeks revealed distinct healing outcomes among the experimental groups (Fig. 7A). The control group exhibited nonunion characteristics, including extensive fibrous tissue infiltration, medullary canal closure, and pseudoarthrosis formation. In the SB group, the implanted material was largely encapsulated by dense fibrous tissue, with only limited bone formation at the defect margins. The PB scaffold promoted substantial osteoconduction, as evidenced by new bone formation extending into the interconnected pore network. Additional incorporation of DPM further enhanced tissue infiltration and defect bridging. The most advanced regeneration was observed in the biomimetic bone group, where abundant newly formed bone occupied the defect region and bridged the host bone ends. Concurrently, extensive scaffold degradation and replacement by mineralized tissue were evident throughout the regenerated area. Higher-magnification examination revealed that regeneration within this hierarchical scaffold proceeded predominantly through endochondral ossification, with clearly identified proliferating chondrocytes, hypertrophic cartilage, and calcified cartilage templates within the bridging zones (Fig. S3)—indicating active cartilage-to-bone conversion during repair. Masson’s trichrome staining further corroborated these observations (Fig. 7B). Extensive mature mineralized bone was observed throughout the regenerated region in the biomimetic bone group, resulting in seamless integration with adjacent host bone. In contrast, less mature collagen-rich tissue and incomplete mineralization were observed in the other treatment groups.

**Fig. 7.**
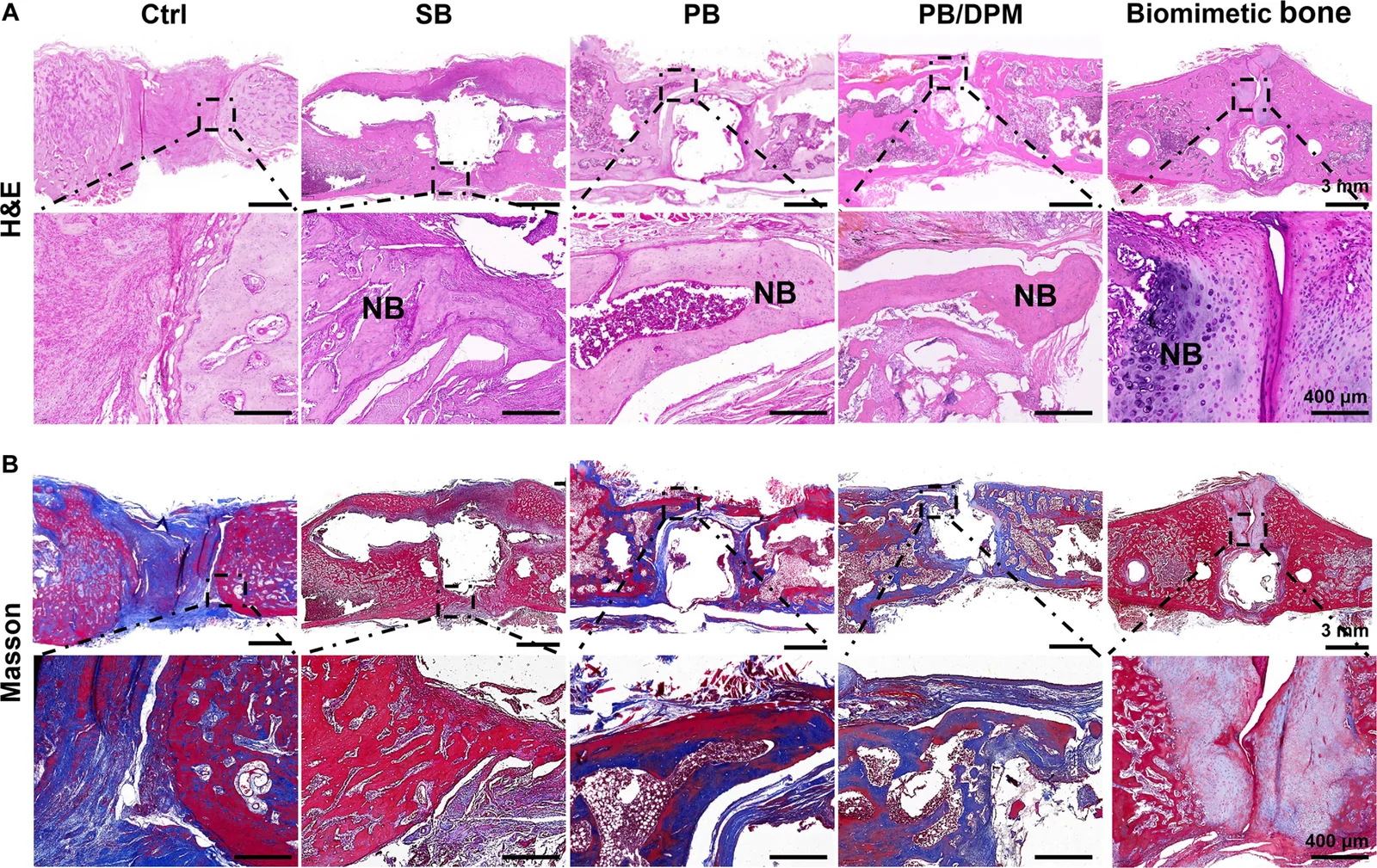
Histological evaluation of long-term bone regeneration. (A) Hematoxylin and eosin (H&E) staining of the defect areas at 16 weeks postimplantation. (B) Masson’s trichrome staining showing collagen deposition and bone maturation. (Note: Red indicates mineralized bone; blue indicates fibrous tissue/collagen). SB, solid brushite; PB, porous brushite; DPM, decellularized periosteal matrix; DBM, decellularized bone matrix; NB, new bone.

### Enhanced angiogenesis and osteogenesis in regenerated bone tissue

Given the critical role of vascularization in large bone defect repair, immunohistochemical analyses were performed to assess angiogenesis and osteogenesis within the regenerated tissues. CD31 staining revealed progressively increasing vascular density from the SB group to the PB group and further to the DPM- and DBM-containing scaffolds (Fig. 8A). The strongest CD31 expression was observed in the biomimetic bone group, indicating extensive neovascularization throughout the defect region. Consistent with the angiogenic findings, expression of the osteogenic markers type I collagen (COL1) and osteopontin (OPN) exhibited similar trends. Enhanced COL1 deposition indicated active ECM synthesis and maturation, whereas elevated OPN expression reflected ongoing mineralization and bone remodeling. Quantitative analysis confirmed significantly greater CD31-positive vessel density as well as larger COL1- and OPN-positive areas in the biomimetic bone group than in all other experimental groups (Fig. 8B). These results demonstrate that the scaffold promotes coordinated angiogenesis and osteogenesis, thereby supporting vascularized bone regeneration and functional restoration of critical-sized segmental defects.

**Fig. 8.**
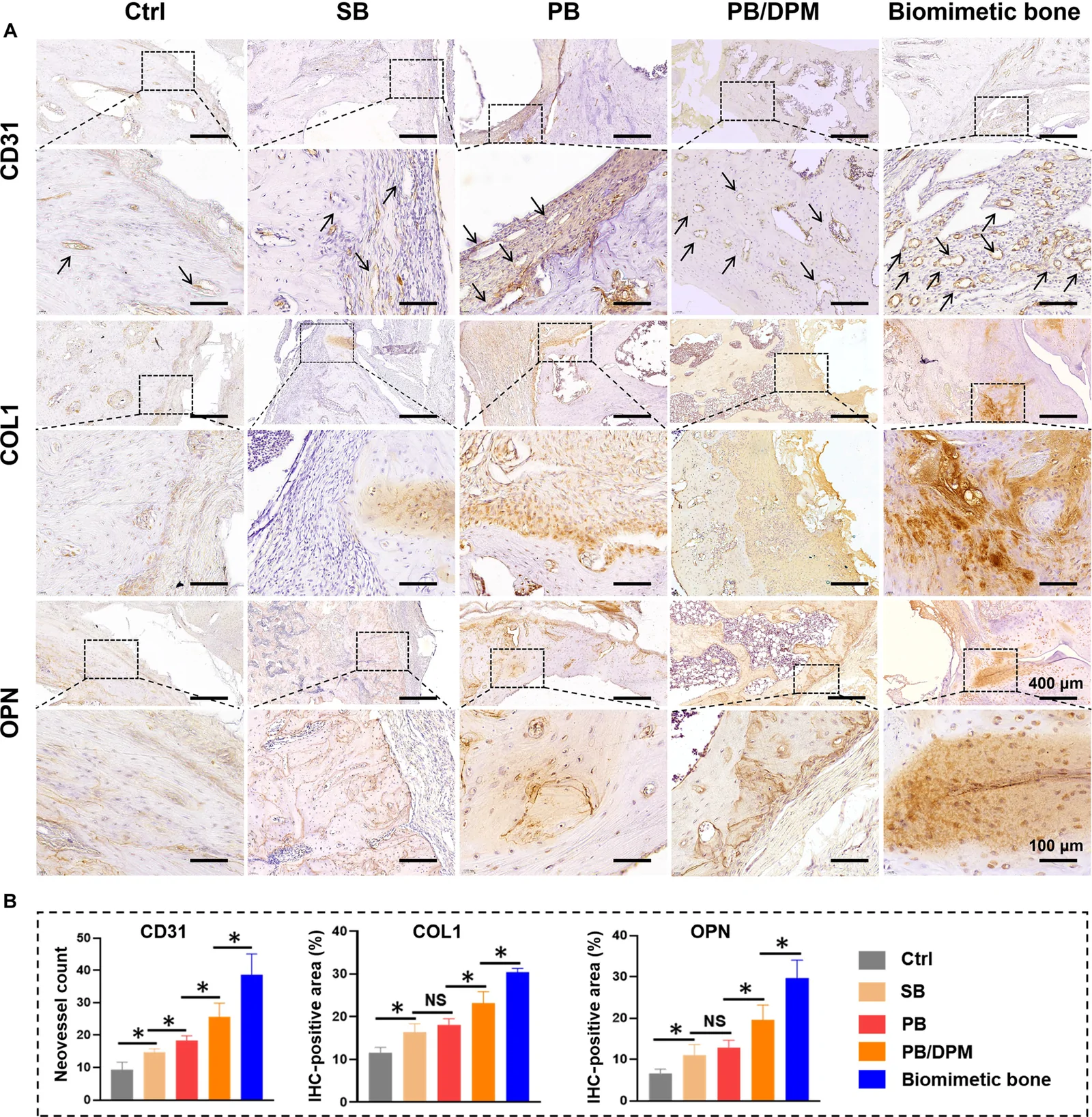
Immunohistochemical (IHC) analysis of vascularized bone tissue. (A) Representative IHC images for CD31 (angiogenesis), type I collagen (COL1), and osteopontin (OPN; osteogenesis) at the defect site; black arrows indicate CD31-positive microvessels. (B) Quantitative analysis of microvessel density and positive staining areas for osteogenic markers. SB, solid brushite; PB, porous brushite; DPM, decellularized periosteal matrix; DBM, decellularized bone matrix. *P < 0.05; NS, no significant difference.

## Discussion

Critical-sized segmental bone defects remain a major challenge in orthopedic reconstruction, as their successful repair requires coordinated restoration of mechanical integrity, vascular supply, and biological function across large defect regions [42,43]. Although the Masquelet technique has become a widely used clinical strategy, its reliance on staged surgery, nondegradable spacers, and delayed grafting inevitably increases patient morbidity and prolongs rehabilitation. The present study sought to address these limitations through the development of a hierarchical biomimetic scaffold that recapitulates the periosteal, cortical, and medullary compartments of native long bone within a single degradable construct. By integrating a DPM, a load-bearing porous brushite framework, and a DBM core, the scaffold provides coordinated structural and biological functions that collectively support the single-stage regeneration of segmental bone defects. A key finding of this study is that reconstruction of the hierarchical bone microenvironment substantially enhanced both osteogenesis and angiogenesis [44,45]. Native long-bone healing is orchestrated through continuous interactions among periosteal progenitor cells, vascular networks, mineralized matrices, and marrow-derived signaling molecules. Conventional synthetic scaffolds generally provide mechanical support but fail to reproduce this biological complexity. In contrast, the present design combines complementary regenerative cues derived from each compartment. The brushite framework provides sustained release of calcium and phosphate ions, which have been reported to stimulate osteogenic differentiation through the activation of pathways including Wnt/β-catenin signaling and subsequent upregulation of RUNX2 [46–48]. Simultaneously, DPM and DBM contribute distinct, complementary biological microenvironments essential for long-bone healing. The outer DPM provides an aligned, dense collagenous fibrous architecture that mimics native periosteum, acting as a biological sleeve to prevent fibrous soft-tissue collapse into the defect while recruiting skeletal progenitors and guiding circumferential neovascularization. In contrast, the inner DBM provides a porous, trabecular template that recreates the endosteal/medullary niche, offering rich adhesive ligands that support central osteoblast lodging and inward bone remodeling. The synergistic interaction of these structural and biochemical signals contributed to the marked enhancement of osteogenic markers, in vitro *vegfa* upregulation (Fig. S2), and robust in vivo CD31-positive vascularization (Fig. 8A and B). Although endogenous signaling molecules (e.g., bone morphogenetic proteins [BMPs] and VEGF) were not directly quantified in our isolated matrices, the established literature demonstrates that detergent-based and enzymatic decellularization partially preserves matrix-bound growth factors within the insoluble ECM, which act synergistically with brushite ion release to activate osteogenic and angiogenic pathways. Concurrently, the progressive resorption of the brushite mineral phase and ECM components actively modulates the immune microenvironment. Natural and synthetic biomaterial cues have been shown to orchestrate macrophage phenotypic transitions towards a prohealing (M2) state, releasing angiogenic and osteogenic cytokines that drive coordinated tissue remodeling [49]. Importantly, the enhanced vascularization observed in the hierarchical scaffold group may be particularly relevant for large segmental defects, where insufficient blood supply frequently results in delayed healing, nonunion, or central tissue necrosis [45].

Beyond biological activity, restoration of mechanical function represents a critical benchmark for successful segmental defect repair. Mechanical failure commonly occurs at the implant–bone interface, where insufficient integration leads to micromotion, fibrous tissue formation, and eventual nonunion [50]. The porous brushite framework was therefore designed to balance mechanical competence with tissue infiltration. The selected architecture generated an interconnected pore network while maintaining sufficient compressive strength to withstand physiological loading in the rat femur. Notably, the incorporation of DPM and DBM further improved mechanical performance beyond that achieved by the porous ceramic alone. High-resolution imaging revealed extensive bone ingrowth throughout the scaffold and intimate contact between regenerated bone and the degrading material surface, without detectable interfacial gaps. These observations indicate true osseointegration rather than passive fibrous encapsulation [51]. Such intimate interfacial osseointegration establishes continuous mineralized bonding between the dissolving brushite framework and host cortices, effectively mitigating interfacial shear stress and preventing micromotion to ensure functional load transmission. Consistent with this interpretation, the hierarchical scaffold exhibited substantially greater resistance to mechanical loading and pullout forces than all control groups (Fig. 6C to G). Together, these findings suggest that reconstruction of periosteal and medullary niches not only enhances bone formation but also facilitates the establishment of a mechanically competent bone–implant interface.

Successful bone regeneration requires a coordinated balance between scaffold degradation and new tissue formation. Excessively rapid degradation compromises mechanical stability, whereas delayed resorption impedes remodeling [52]. Although brushite is more resorbable than many calcium phosphate ceramics, achieving optimal coupling between degradation and regeneration remains challenging. In this study, scaffold degradation proceeded in parallel with progressive bone formation, ultimately enabling extensive replacement of the construct by new bone (Fig. 7). The incorporation of decellularized matrices likely facilitated this process by enhancing cellular infiltration and promoting the recruitment of osteoclasts and macrophage-lineage cells involved in material resorption [53]. Consequently, the scaffold functioned as a transient regenerative template rather than a permanent implant, enabling a continuous scaffold-to-bone transformation that minimizes long-term foreign-body retention while maximizing tissue restoration.

From a translational perspective, the hierarchical scaffold offers several advantages over current clinical strategies. It eliminates donor-site morbidity associated with autologous bone grafting and avoids the need for a second surgery required in the Masquelet technique [54]. In addition, the construct can be prefabricated as an off-the-shelf implant that simultaneously provides mechanical support, osteoinductive cues, and controlled biodegradation. Nevertheless, limitations remain, including the lack of full biomechanical fidelity in the rat femoral model and incomplete understanding of the molecular mechanisms underlying DPM- and DBM-mediated regeneration [55,56]. Although the insoluble collagen network was well retained to support cell attachment and tissue ingrowth, chemical decellularization and freeze–thaw cycles inevitably led to the partial depletion of soluble glycosaminoglycans. In addition, given the biochemical complexity and trace quantities of endogenous signaling molecules within native bone and periosteum, quantitative profiling of specific growth factors (such as BMP-2 and VEGF) was not conducted in this study. Furthermore, while qualitative micro-CT and histological evaluations demonstrated progressive scaffold resorption alongside robust bone ingrowth, quantitative, longitudinal degradation kinetics (such as dynamic mass loss and volumetric resorption rates) were not explicitly tracked to verify kinetic matching. Finally, inherent geometric variations among individual rat femora represent an inevitable source of variation in ex vivo biomechanical assessments. Future studies should therefore focus on in-depth proteomic validation and evaluation in large-animal load-bearing models under clinically relevant loading conditions. Despite these limitations, this work demonstrates that hierarchical reconstruction of bone microenvironments provides a promising strategy for the single-stage repair of critical-sized segmental defects.

## Conclusion

In conclusion, we developed a hierarchical triphasic biomimetic scaffold that recapitulates the periosteum–cortical bone–cancellous bone organization of native long bone and enables the single-stage repair of critical-sized segmental defects. By integrating a porous brushite framework with decellularized periosteal and bone matrices, the construct provides a synergistic combination of mechanical stability, osteoinductive signaling, and angiogenic support. In vivo results demonstrate that this hierarchical design promotes rapid vascularized bone regeneration, robust osseointegration, and functional biomechanical recovery while eliminating the need for secondary implant removal. The observed regenerative process is characterized by coordinated osteogenesis and endochondral ossification within a progressively degrading scaffold. This work demonstrates an effective strategy for the single-stage repair of load-bearing segmental bone defects. It further highlights the potential of hierarchical biomimetic scaffolds for complex bone reconstruction.

## Materials and Methods

### Preparation of the hollow porous brushite framework

#### Preparation of microspheres

To construct an interconnected macroporous architecture, sacrificial un-cross-linked gelatin microspheres (200 to 400 μm) were synthesized via a water-in-oil emulsion technique. Briefly, a 10 wt% gelatin solution (Sigma-Aldrich, USA) was prepared at 50 °C. Ten milliliters of this solution was added dropwise into 50 ml of soybean oil under continuous magnetic stirring (800 rpm, 60 °C) for 10 min and then rapidly cooled in an ice bath to solidify the droplets. The microspheres were thoroughly washed 3 times with acetone to extract the oil phase, followed by 3 washes with absolute ethanol for dehydration. After vacuum drying, microspheres with diameters of 200 to 400 μm were harvested using 45-mesh and 80-mesh sieves.

#### Preparation of solid brushite

The mineral phase of the scaffold, DCPD (brushite), was synthesized by mixing β-tricalcium phosphate and monocalcium phosphate monohydrate (Sigma-Aldrich, USA) at a mass ratio of 0.55:0.45. A 0.5 M citric acid (Sigma-Aldrich, USA) solution was utilized as the liquid phase, with a liquid-to-powder ratio of 250 μl/g. The liquid phase was thoroughly mixed into the solid phase of the cement, and the obtained cement paste was then filled into polydimethylsiloxane molds.

#### Preparation of the hollow porous brushite framework

Gelatin microspheres were incorporated into the cement paste at varying weight fractions (5%, 10%, and 15%). After preliminary screening based on mechanical strength, 10 wt% gelatin microspheres were incorporated into the cement paste as an optimized formulation to achieve a balanced compressive strength of 11.0 MPa, which meets the critical in vivo load-bearing requirements (1.0 MPa) and dynamic ambulation demands of the rat femoral defect model. The mixture was cast into customized cylindrical polydimethylsiloxane molds. After initial setting, the constructs were immersed in deionized water at 55 °C for 24 h to completely leach out the un-cross-linked gelatin templates via thermal dissolution, resulting in a hollow porous brushite (PB) framework.

### Fabrication of decellularized matrices (DBM and DPM)

To recapitulate the biological microenvironments of bone marrow and periosteum, DBM and DPM were prepared [57]. DBM preparation: Fresh porcine cancellous bone was harvested, crushed into granules, and decalcified in 0.5 M HCl for 24 h. Lipids were removed using a chloroform/methanol mixture (1:1 v/v) solution, followed by enzymatic treatment with 0.05% trypsin and 0.02% EDTA to eliminate cellular components. DPM preparation: Porcine periosteum was isolated and subjected to 3 freeze–thaw cycles. The tissues were then treated with 2% Triton X-100 for 12 h and 100 U/ml DNase I for 12 h to achieve complete decellularization while preserving the ECM architecture. To validate decellularization efficacy, histological sections were stained with H&E and DAPI to qualitatively confirm the complete clearance of intact cells and visible nuclear fragments. In addition, total residual double-stranded DNA (dsDNA) was extracted and quantified using a Quant-iT PicoGreen dsDNA assay kit (Invitrogen, Carlsbad, CA, USA) according to the manufacturer’s instructions; a residual DNA level below 50 ng/mg dry tissue weight and fragment sizes under 200 bp confirmed thorough decellularization [58].

### Assembly of the hierarchical biomimetic scaffold

The biomimetic scaffold was constructed by spatially organizing 3 functional compartments. First, DPM was hydrated in sterile phosphate-buffered saline (HyClone, USA) and wrapped around the outer surface of the PB framework to serve as a periosteum-mimicking layer. Second, DBM granules were introduced into the central cavity of the PB framework to simulate the medullary microenvironment. Prior to in vivo implantation, the assembled constructs were vacuum-sealed in medical pouches and terminally sterilized by ^60^Co γ-irradiation (25 kGy, low temperature, Irradiation Center of Suzhou University, Suzhou, China). This protocol ensures complete sterility while preserving ECM bioactivity and scaffold architecture.

### Structural and mechanical characterization

Samples were fixed onto stubs with conductive tape and sputter-coated with gold for 45 s. The morphology was imaged via SEM (FEI Company, USA) at 15 kV, while the elemental composition was quantified through concurrent energy-dispersive x-ray spectroscopy point-scan analyses. Biomechanical evaluation (tensile pullout strength) of explanted femora was conducted on a universal testing machine at a crosshead speed of 1 mm/min. Following careful removal of the fixation hardware, both proximal and distal bone ends were secured in custom self-aligning pneumatic grips with a standardized gauge clamping distance to minimize axial misalignment and shear artifacts.

### In vitro osteogenic and angiogenic evaluation

BMSCs were utilized to assess the bioactivity of the scaffolds. Cell proliferation and viability were determined using CCK-8 (Kumamoto, Japan) and Live/Dead assays (Carlsbad, CA, USA). For osteogenic evaluation, BMSCs were cultured in a Transwell co-cultured system with scaffolds. ALP activity and ARS staining were performed at 7 and 14 d, respectively. The expression of osteogenic markers (*Alpl*, *Runx2*, *Col1a1*, and *Opn*) and the angiogenic marker (*Vegfa*) was quantified via RT-qPCR. Primer sequences are provided in Table S1. Relative gene expression was calculated using the 2^−ΔΔCt^ method.

### In vivo evaluation in a rat femoral segmental defect model

All animal experimental procedures were approved by the Animal Care and Use Ethics Committee of Soochow University (Approval No. SUDA20230605A04) and conducted in accordance with the national guidelines for laboratory animal care. A 5.0-mm critical-sized segmental defect was created in the mid-diaphyseal region of the right femur using a custom surgical oscillating saw guide under sterile conditions. The hierarchical construct (5.0 mm in length) was press-fitted into the defect gap, and the outer DPM sleeve was circumferentially secured to adjacent host periosteal tissues using surgical sutures. Rigid internal fixation was applied using a customized 4-hole stainless-steel plate fixed with 4 bicortical screws to maintain mechanical alignment during ambulation. Following stable implantation, the muscle layers and skin were closed layer by layer using resorbable sutures. Animals were randomly assigned to 5 groups: (a) control (empty defect), (b) solid brushite (SB), (c) porous brushite (PB), (d) PB/DPM, and (e) biomimetic bone (PB/DPM/DBM). At 16 weeks postsurgery, femurs were harvested for micro-CT analysis (SkyScan, Sweden, 100 kV, 100 mA, and a 1.0-mm Al filter), including BV/TV and Tb.N. Three-dimensional reconstructions and morphometric analyses were conducted using the NRecon, DataViewer, CTAn, and Mimics 15.0 software.

### Histological and histomorphometric analysis

Harvested samples were fixed in 10% neutral buffered formalin, decalcified in 15% EDTA (pH 7.4) for approximately 45 d, and embedded in paraffin. Longitudinal sections (6 μm) were stained with H&E and Masson’s trichrome to evaluate the quality of regenerated bone and the integration between the material and host tissue. Immunohistochemical staining was performed to evaluate in situ osteogenesis and neovascularization. Following antigen retrieval and blocking with 3% bovine serum albumin for 30 min, sections were incubated overnight at 4 °C with primary antibodies against CD31 (1:200; ab28364, Abcam), OPN (1:200; ab13418, Abcam), and COL1 (1:250; ab34710, Abcam). After being rinsed with phosphate-buffered saline, the sections were treated with horseradish peroxidase-conjugated secondary antibodies (1:1,000; Beyotime Biotechnology) for 1 h at room temperature.

### Statistical analysis

Data are presented as mean ± standard deviation. Statistical analysis was performed using the GraphPad Prism 10.0 software. Normality was verified by the Shapiro–Wilk test. Two-group comparisons were performed using a Student *t* test, and multiple comparisons were performed using 1-way or 2-way analysis of variance followed by Tukey’s post hoc test. A *P* value <0.05 was considered statistically significant.

## Data Availability

The data underlying the results presented in this paper are not publicly available at this time but may be obtained from the corresponding authors upon reasonable request and under a licensing agreement.
