# Supplementary material for: A Biomimetic Scaffold for Single-Stage Reconstruction of Load-Bearing Segmental Bone Defects
Source: BME Front. 2026 Oct 8;7:0321. doi: 10.34133/bmef.0321 (PMC13646458; doi:10.34133/bmef.0321)
Supplement: Supplementary file 1 [file bmef.0321.f1.docx]

**A Biomimetic Scaffold for Single-Stage Reconstruction of Load-Bearing Segmental Bone Defects**

Jianzhong Bai^1,2#^, Dachuan Liu^1#^, Jiaying Li^1^, Huan Wang^1^, Guoping Liu^1^, Youzhi Hong^1^, Wenyu Duan^1^, Jiaxu Shi^1^, Hongtao Zhang^1^, Bin Li^1,3^, Jing Qu^4*^, Song Chen^1*^

**Table S1 Primer sequences for RT-qPCR**

| Gene | | **Forward (5′→3′)** | **Reverse (5′→3′)** |
| --- | --- | --- | --- |
| *Alpl* | TGACCGTCCTGCTGGAACTCG | | CCACTGCCACACTTGTCACAGAG |
| *Col1a1* | TGTTGGTCCTGCTGGCAAGAATG | | GTCACCTTGTTCGCCTGTCTCAC |
| *Gapdh* | AGAAGGCTGGGGCTCATTT | | AGGGGCCATCAGTCTTC |
| *Opn* | GACGATGATGACGACGACGATGAC | | GTGTGCTGGCAGTGAAGGACTC |
| *Runx2* | AACAGCAGCAGCAGCAGCAGCAG | | GCACGGAGCACAGGAAGTTGG |
| *Vegfa* | CGGTGTGGTCTTTCGTCCTTCTTAG | | TCGAAGATGGGCTTTGCAGT |

**Fig. S1.** Quantitative evaluation of extracellular matrix (ECM) components in native tissues and decellularized matrices. (A) Total collagen quantification of native periosteum and DPM. (B) GAG content quantification of native periosteum and DPM. (C) Total collagen quantification of native decalcified cancellous bone and DBM. (D) GAG content quantification of native decalcified cancellous bone and DBM. *, *p* < 0.05; NS, no significant difference.

**Fig. S2.** RT-qPCR analysis of pro-angiogenic gene (*Vegfa*). *, *p* < 0.05; NS, no significant difference.

**Fig. S3. High-magnification histological evaluation of endochondral ossification in the biomimetic bone group at 16 weeks.** (A) Magnified view of Fig. 6A showing the calcified cartilage template zone undergoing mineralization (black arrows). (B) Detailed view displaying active chondrogenesis characterized by proliferating and hypertrophic chondrocytes (black arrows).
